# Dr. Brown-Séquard’s Lectures

**Published:** 1878-03

**Authors:** 


Dr. Brown-Séquard, who is too well known to need an introduction to the American medical profession, is at present in this country to visit the larger cities and entertain his professional friends with his observations and researches in that most interesting, yet still most obscure field of medical science, the diseases of the brain. In the past month he paid a visit to our city, and under the auspices of the Chicago Medical Press Association, delivered three lectures on "Paralysis and convulsions as effects of diseases of the base of the brain." We thought that these lectures, embodying the views of one of the greatest authorities on the functions of the brain, would be of more than a passing interest, and deserved to be reproduced in the pages of a medical journal. We therefore secured a complete short-hand report of the lectures, and are pleased to present to our readers the first lecture in this number.

The second lecture will be published in the Journal and Examiner for April, and the third in the issue for May.

